# Engineering bacterial cell morphology for the design of robust cell factories

**DOI:** 10.1016/j.bbrep.2025.102076

**Published:** 2025-06-07

**Authors:** Maarten Lubbers, Nova Jaspers, Dennis Claessen

**Affiliations:** Microbial Sciences, Institute of Biology, Leiden University, PO Box 9505, 2300 RA, Leiden, the Netherlands

**Keywords:** Synthetic biology, Morphology engineering, L-forms, Wall-deficient bacteria

## Abstract

Bacteria come in a wide variety of shapes, ranging from spherical or rod-shaped unicellular cells to complex multicellular structures. These shapes have evolved to benefit the organism in its natural environment. However, industry often takes such organisms from their natural environment to produce useful molecules that favor mankind. Their natural morphology is often far from optimal for use in an industrial setting. Filamentous bacteria, for instance, have a morphology that presents unique challenges for industrial settings. Therefore, various engineering approaches have been developed to optimize their morphology. This review explores a spectrum of successful engineering strategies, offering insights and providing inspiration for future advancements. It holds the potential to lead the way in optimizing morphology in challenging microorganisms and thus improve their exploitability in biotechnology.

## Highlights

• Various morphology engineering approaches have been developed to optimize cell factories.
• New approaches are required to facilitate significant redesigns of cell morphology.
• L-forms present a promising and innovative opportunity to engineer morphologies.
• *Streptomyces* L-forms could potentially be used to develop unicellular cell factories.

## 1 Introduction

Microbes are used extensively to produce high-value biochemical products, such as antibiotics, anticancer agents, and enzymes [[1], [2], [3], [4]]. Choosing a microbial production host is often dependent on its tractability, as species are favored that are easily culturable and grown on a large scale, with well-studied genome sequences and metabolic pathways, and are feasible for genetic manipulation [5,6]. Well-known, canonical examples include the bacteria *Bacillus subtilis* [7], *Escherichia coli* [8], and the fungi *Aspergillus niger* [9], *Pichia pastoris* [10] and *Saccharomyces cerevisiae* [11].

In bacteria, recent strategies for improving industrial production have mostly focused on engineering metabolism by, for instance, designing synthetic pathways, enabling researchers to plug in and plug out genetic circuits and regulatory devices [5,[12], [13], [14]]. At the same time, non-canonical species – species which have not been well-established yet as production platforms - are being investigated, such as *Roseobacter denitrificans* [15], *Deinococcus radiodurans* [16] and *Methylobacterium extorquens* [17]. Cyanobacteria are also of large interest due to improved engineering strategies and their ability to grow on carbon dioxide, aided by light [18]. Despite various advancements, such as applying codon optimization tools, novel genetic engineering methods, and multi-omics approaches [19], integrating non-canonical species for industrial applications remains challenging, resulting in only a limited number of microbial cell factories being exploited to date [5]. One other area that remains largely unexplored is the potential advantage of modified and optimized bacterial shapes for improving production processes [20], presumably, because many genes involved in cell shape determination are essential [21]. Bacteria display a plethora of morphologies, such as rods, cocci, or multicellular hyphae. These shapes are the result of selective pressure affecting, amongst others, nutrient acquisition, motility, and interactions with other organisms [22]. However, their natural morphology is often far from optimal for use in an industrial setting [23]. In this review, we will primarily delve into exploring how engineering morphology is a promising field of research for improving cell factories. Moreover, we will present a groundbreaking approach aimed at addressing constraints associated with engineering morphology through the substitution of “essential” shape-defining genes, thereby possibly paving the way to broaden the range of host organisms.

## 2 Morphology regulation in uni- and multicellular bacteria

In bacteria, the cell shape is dictated by the cell wall, which serves as a protective barrier surrounding the cells [24,25]. The cell wall architecture can vary between different bacteria. Where monoderm bacteria generally have a thick peptidoglycan (PG) layer, diderm bacteria have one PG layer placed between the cytoplasmic and the outer membrane [26]. PG consists of strands of alternating N-acetylglucosamine and N-acetylmuramic acid residues. These chains are intricately cross-linked through peptide bridges between neighboring N-acetylmuramic acid units. Synthesis of PG is mediated by two distinct polymerase systems [27]. Shape, elongation, division, and sporulation (SEDS) transglycosylases synthesize the PG strands [28], whereas class B penicillin binding proteins (bPBP) transpeptidases catalyze the cross-linking reactions [29]. These complexes are intracellularly positioned by cytoskeletal proteins. In unicellular bacteria, the so-called MreB system is involved in cell elongation [30], while the FtsZ system is required for PG synthesis during cell division [31]. Mutations in genes related to the MreB system typically lead to cells with a change in the cell diameter [32,33], while mutations in cell division genes typically yield cells with a changed length [34]. One important aspect is that the proteins operating in the elongation or division machinery are oftentimes essential and can therefore not be deleted [35,36]. Therefore, CRISPRi approaches have been developed to tune the expression of these essential genes [37,38].

**Fig. 1:**
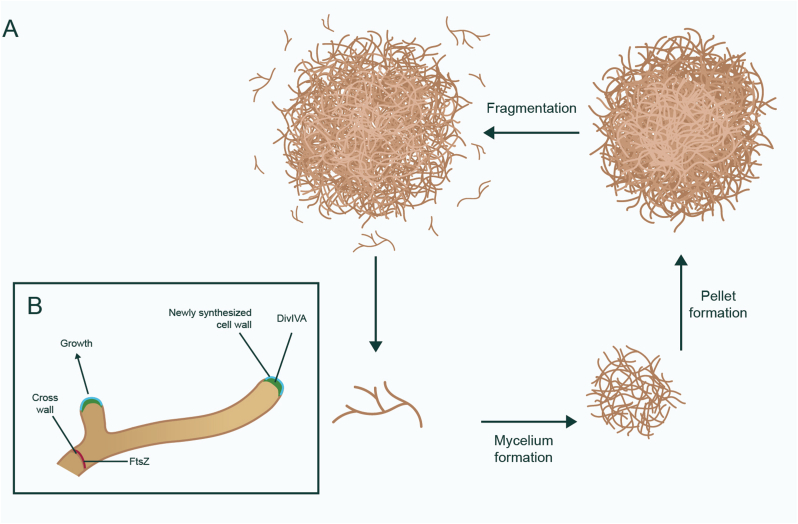
**an overview of morphogenesis in multicellular bacteria.** A) Streptomycetes are bacteria that form large multicellular networks of hyphae, called mycelia. Such hyphae are compartmentalized by the formation of cross-walls, which is orchestrated by the FtsZ protein. Elongation and branching of hyphae are coordinated by DivIVA which resides at growing tips. B) In bioreactors, mycelia can grow and aggregate to form large, dense pellets. During the stationary phase of growth, pellets disintegrate and release smaller fragments, which can then grow into new pellets.

Tight cooperation between the elongation and division machineries ensures that most unicellular bacteria grow in a largely predictable manner. Following a period of elongation, cells divide and separate to form two identical daughter cells. This is in stark contrast to multicellular bacteria, such as streptomycetes, which are filamentous bacteria widely used in industry to produce antibiotics, enzymes, and anticancer agents [[39], [40], [41]]. Streptomycetes form long interconnected filaments, called hyphae, that do not separate after cell division (Fig. 1A). They grow by tip extension, a process that is coordinated by the DivIVA protein (Fig. 1B) [42]. DivIVA interacts, amongst others, with the cytoskeletal proteins Scy and FilP, to form a dynamic and crucial complex at the tip regulating the insertion of new cell wall material [[43], [44], [45]]. While the PG-synthesizing enzymes in *Streptomyces* have not been identified yet, DivIVA also interacts with CslA, a cellulose synthase-like protein that is involved in the biosynthesis of β-glucan at mycelial tips [[46], [47], [48]]. Cell division, on the other hand, is orchestrated by FtsZ, leading to the formation of cross-walls within the hyphae (Fig. 1B). The filaments occasionally branch, leading to the establishment of new growth sites [49]. By combining division, elongation, and branching, an entangled network of filaments is established, called a mycelium, comparable to that formed by filamentous fungi. Some hyphae are degraded after programmed cell death and the remaining viable hyphae undergo compartmentalization [50,51], which leads to the formation of specialized hyphae that protrude into the air and their subsequent development into chains of unigenomic spores.

## 3 Correlation between cell morphologies and downstream processing

Multicellular bacteria are widely used in industry to produce a diverse range of complex compounds [52]. For example, streptomycetes are well-known for the production of various antibiotics, such as streptomycin [53], antifungal compounds such as natamycin [54] and nystatin [55], and numerous anti-cancer compounds like doxorubicin [56] and landomycin [57]. In addition, streptomycetes are used for producing industrial-relevant enzymes such as tyrosinases [58], inulinase [59], and glucose isomerase [60]. These bacteria have potent secretion systems via which products are directly released into the medium. This is an important advantage for industrial production because it simplifies downstream processing and may help solve toxicity and production issues, with higher yield as an attractive perspective [61].

The mycelial mode of growth can negatively impact industrial processing, as it causes slow growth, high viscosity of the culture broth, and morphological heterogeneity in cultures, making them less favorable from a fermentation perspective [[62], [63], [64]]. For instance, mycelia can aggregate to form dense structures called pellets [65] (Fig. 1A). These pellets can exceed 200 μm in size, thereby limiting efficient nutrient and oxygen transfer, restricting growth [66,67]. This aggregation is caused by extracellular glycans that act as glue-like substances [68]. Well-known glycans are poly-β-1,6-N-acetylglucosamine (PNAG) produced by the *matAB* locus and the cellulose-like glycan produced at hyphal tips [65,68,69].

Moreover, the effective production of metabolites often correlates with a specific morphology. For instance, pellet formation is necessary for nikkomycin production in *Streptomyces tendae* [70], erythromycin in *Saccharopolyspora erythraea* [66], and tylosin production in *Streptomyces fradiae* [71], all stimulated by oxygen and nutrient shortage, while in other cases, pellet formation impairs nystatin production in *Streptomyces noursei* [72]. For enzyme production, the formation of pellets is generally unfavorable, and instead, a preference is given to more dispersed and open-growing mycelia. As nutrients are more easily accessible to all hyphae, this leads to faster growth and increased enzyme production [65,73]. For instance, fragmentation of mycelial clumps resulted in an increase in tyrosinase production in *Streptomyces lividans* [73]*.* These examples correlate specific morphologies with their impact on industrial productivity. Furthermore, processes during industrial fermentation, such as agitation, aeration, and pH levels, can cause hydromechanical stress, and influence oxygen and nutrient transfer [74]. For an in-depth analysis of the physiological responses of streptomycetes to these conditions, we would like to refer to a review by Olmos et al. [74].

One way to overcome these challenges is to produce specialized metabolites or enzymes heterologously using a unicellular host. Advances in synthetic biology have made it possible to clone biosynthetic gene clusters directly into preferred production hosts. For example, *B. subtilis* has been used to express 6-deoxyerythronolide B (6dEB) from *Saccharopolyspora erythraea* [75]. This included cloning the deoxyerythronolide B synthase (DEBS) gene cluster, consisting of three different proteins. Additionally, 6dEB production was increased when the production of surfactin, bacillaene, and plipastatin was inhibited. In other research, enniatin from *Fusarium oxysporum* was produced in *B. subtilis* using an inducible promoter system [76]. When the cultivation conditions were optimized, this resulted in secretory production of enniatin B [76]. *Corynebacterium glutamicum* was used for the heterologous production of the antibiotic roseoflavin from *Streptomyces davaonensis* [77]. This included the expression of three roseoflavin biosynthetic genes, namely *rosB*, *rosA* and *RFK* [77].

Although these hosts hold significant promise for generating relatively simple molecules, the heterologous production of complex antibiotics, which are typically synthesized through the concerted action of numerous proteins, seems to pose a considerable challenge [78,79]. Furthermore, proteins from streptomycetes often have post-translational modifications or folding requirements that differ from those in the heterologous host, further complicating their expression and production [61,80,81]. To conclude, the production of these molecules is currently restricted to closely related species. Therefore, we need to have a better understanding of how to control their morphology.

## 4 Advancements in morphology engineering in multicellular bacteria

**Fig. 2:**
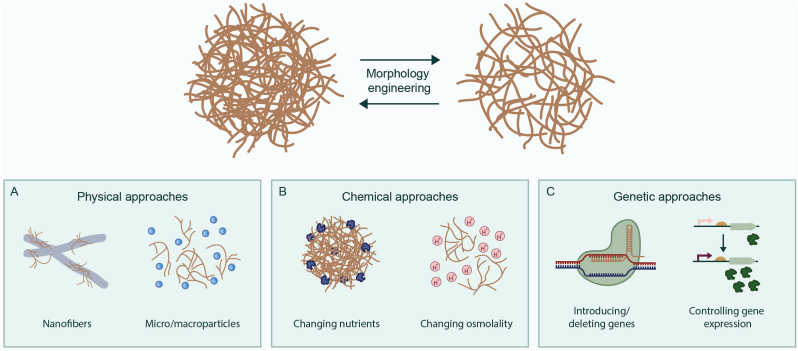
**Overview of the three central approaches to engineer the morphology of multicellular bacteria.** Physical approaches include the use of nanofibers and micro- and macroparticles (A). Chemical approaches include changing nutrients and medium osmolality (B). Genetic approaches include deleting and introducing genes and controlling gene expression (C).

Various physical approaches have been investigated to optimize the morphology of multicellular bacteria in industrial settings (Fig. 2A). For instance, the addition of charged polymers, such as junlon, prevented initial aggregation of mycelia of streptomycetes [82]. Charged polymers likely interfere with the electrostatic characteristics of the cell wall, thereby inhibiting the initial aggregation process [83]. Microparticles have also been applied in streptomycetes. For example, the addition of talc powder microparticles decreased the average pellet size of *Streptomyces albus* sixfold, while enhancing the production of pamamycin [84]. Likewise, the addition of glass particles to *S. coelicolor* cultures improved actinorhodin production by more than 85 % [85]. The presence of glass particles facilitated the transformation of pellet morphologies into dispersed mycelia, enhancing oxygen transfer and boosting the production of specialized metabolites [86]. In other research, the addition of microparticles to *Streptomyces rimosus* cultures caused, amongst others, the deformation of pellets and an increase in size variation. Besides, an increase was seen in specialized metabolite production, such as oxytetracycline [87]. The addition of microparticles has also been used in filamentous fungi. In *A. niger*, the introduction of macroparticles hindered the formation of pellets, resulting in the formation of dispersed mycelial structures and loose clumps [88]. In *Caldariomyces fumago*, microparticles led to the mycelium dispersion up to the level of single hyphae, increasing the production of chloroperoxidase [89].

In addition to beads, organic nanofibers have been used to alter the morphology of streptomycetes. In liquid cultures, these fibers could function as an extra filamentous scaffold, expanding surface availability for mycelial growth [90]. In *S. lividans*, the addition of nanofibers caused an increased production of actinorhodin and other antibiotics [90]. Besides, the type of impellers, as well as the stirring speed, can have a strong influence on the morphology of multicellular microbes in bioreactors [91]. Hereby, a higher agitation speed produces smaller pellets, which are metabolically different than larger pellets [92,93]. At low stirrer speeds, gas is inadequately dispersed, limiting the sufficient transfer of oxygen. In some cases, this can increase antibiotic production [67]. Furthermore, alternative cultivation systems are also being explored further to circumvent aggregation, such as the use of microtiter plates [86,94]. Collectively, these examples show how physical forces imposed by altering stirring speeds or the addition of nanoparticles can alter productivity in filamentous microbes.

Chemical approaches have also been used for morphology engineering in streptomyces (Fig. 2B). For instance, changing medium composition has been shown as a valuable method for morphology engineering. For instance, the addition of galactose, ammonium sulfate, and copper caused a significant reduction in pellet size in *Streptomyces toxytricini*. However, this also reduced the formation of biomass [95]. Furthermore, the addition of sodium nitrate inhibited growth in *S. noursei*, reducing biomass and increasing oxygen uptake [72]. Lastly, extracellular substances like proteins, sugars, and DNA could also act as adhesive extracellular polymers, possibly stimulating the formation of pellets [96]. In multicellular fungi, chemical approaches have been applied as well. For instance, in *A. niger*, an increase in osmolality led to a more homogenous culture with more elongated filaments. Further increasing the osmolyte concentration resulted in the complete cessation of pellet formation [97].

In addition to physical and chemical methods, genetic approaches have been pursued to alter the morphology of streptomycetes (Fig. 2C–Table 1). Activating the cell division-stimulating protein SsgA increased mycelial fragmentation, thereby reducing fermentation times and enhancing productivity [73]. Likewise, deletion of either *matA* or *matB* led to a phenotype with very small, open mycelia [68,69], coinciding with an increased growth rate of these mutants by 60 % compared to the wild-type strain [68]. Two-component systems have also been investigated as a target for morphology engineering. A single amino acid substitution in the Sco5282 kinase, which has a cognate response regulator called Sco5283, indirectly resulted in slow-sedimenting pellets in *S. coelicolor* [98]. This change in pellet morphology was more likely to be caused by one of the 24 transcriptional regulators differently expressed by the two-component system [98]. In addition to the methods mentioned above, directed evolution and high-throughput mutant screening offer valuable complementary strategies. For example, prolonged selection in a chemostat for over 100 generations led to the discovery of a non-pelleting phenotype in *S. lividans* [99]. In fact, this phenotype was later discovered to be caused by mutations in the *matA* and *matB* genes [69].

**Table 1:** Genes utilized in bacterial morphology engineering strategies.

Gene	Function	Host	Uni-/Multicellular	Type of manipulation	Target process	Effect on phenotype
*mreB*	Cell shape regulation	*S. elongatus* [114]	Unicellular	Partial deletion; overexpression	Downstream processing	Spherical cells; spindle-shaped cells
		*S. elongatus* [114]	Unicellular	Partial deletion; overexpression	Downstream processing	Spherical cells; spindle-shaped cells
*sulA*	Cell division inhibitor	*E. coli* [110]	Unicellular	Overexpression	PHA production	Filamentous cells
*ftsZ*	Septum localization	*H. campaniensis* [112]	Unicellular	Decreasing expression	PHB production	Cell size expansion; cell shape elongation; cell gravity precipitation
		*S. elongatus* [114]	Unicellular	Inhibiting expression; overexpression	Downstream processing	Filamentous growth; shorter cells
*min system*	Septum localization	*S. elongatus* [113]	Unicellular	Overexpression	Downstream processing	Increased cell length
*rodA*	Cell shape and length regulation	*S. elongatus* [114]	Unicellular	Partial deletion	Downstream processing	Spherical cells
*ssgA*	Cell division-stimulating protein	*S. coelicolor* [73]; *S. lividans* [73]	Multicellular	Activation	Mycelial fragmentation	increased mycelial fragmentation
*matA* and *matB*	Synthesis of extracellular glycans	*S. coelicolor* [68]; *S. lividans* [68,69]	Multicellular	Deletion	Mycelial fragmentation	Small and open mycelia
*sco5282*	Kinase (indirect effector)	*S. coelicolor* [98]*, S. lividans* [98]	Multicellular	single amino acid substitution	Pellet formation	Slow-sedimenting pellets

It is important to highlight that changing mycelial morphologies can impact growth or yield if overdone. For instance, a smaller pellet size can be beneficial for downstream processing, however, this directly results in a lower biomass [100]. Besides, this could also cause excessive fragmentation of mycelia, reducing specialized metabolite production [66,70]. Therefore, when applying the above-mentioned morphology engineering techniques, it is important to balance the benefits of optimized cell shape for downstream processing with the overall impact on process performance.

## 5 Advancements in morphology engineering in unicellular bacteria

Unicellular bacteria, such as *E. coli* and *C. glutamicum*, are often preferred hosts for industrial production. These organisms are industrially exploited to produce, amongst others, therapeutic proteins, vaccines, and amino acids [[101], [102], [103]]. Compared with multicellular bacteria, unicellular bacteria are often more predictable to cultivate in large-scale industrial settings [62,63]. Besides, unicellular bacteria are also often amenable to genetic engineering techniques, enabling the modification of their metabolic pathways for enhanced product yields or the production of novel compounds [104,105]. Despite these benefits, production processes can sometimes be optimized by using cells with an increased size. For instance, small cell sizes can be a limiting factor in producing intracellular products. The best-known example where cellular sizes were increased to improve production is related to the production of polyhydroxyalkanoates (PHA) by bacteria, which accumulate inside intracellular inclusion bodies [[106], [107], [108]]. These biopolyesters have a large potential to substitute petroleum-based plastics, as they are both biodegradable and biocompatible [109]. Different approaches have been undertaken to increase PHA production in bacteria (Table 1). For instance, overexpression of the cell division inhibitor SulA caused the formation of filamentous *E. coli* cells and strongly increased the accumulation of inclusion bodies [110].

*Halomonas campaniensis* has been of interest for PHB production as well [111]. To enhance the capacity for inclusion body formation, the morphology of *H. campaniensis* was engineered by modulating the expression of *mreB* and *ftsZ*, as larger cell sizes are more suitable for accommodating these structures [112]. This was done via a temperature-responsive plasmid expression plasmid. By first growing temperature-sensitive *mreB* or *ftsZ* mutants at 30 °C till a certain cell density, expression levels were induced at 37 °C causing cell size expansions or cell shape elongations [112]. Furthermore, the changed morphologies also caused cell gravity precipitation, helping with cell separation and thus downstream processing.

Morphological manipulation has been a focus for unicellular cyanobacteria as well. Cyanobacteria hold great promise for producing fuels, chemicals, and biomass, but challenges remain in their harvesting and processing [113]. Increasing cell length is a key strategy, as larger cells are easier to collect [113]. Therefore, in *Synechococcus elongatus*, the cell length was extended from several micrometers to near-millimeter lengths by expressing different components of the Min system, which orchestrates the localization of the septum prior to cell division [113]. Interestingly, the elongated cells exhibited an increased sedimentation rate and were more susceptible to lysis, possibly decreasing harvesting and processing costs [113]. In other research, FtsZ defective mutants of *S. elongatus* grew filamentous, while overexpression of FtsZ resulted in shorter cells [114]. When MreB and RodA, a transglycosylase involved in cell shape and length regulation, were partially deleted, cells transformed from rod-shaped to spherical [114]. Conversely, overexpression of MreB elicited spindle-shaped cellular morphologies. Experimental evolution and high-throughput screens are also relevant strategies for screening morphology-enhanced mutants. For instance, changes in cell size and shape were found in a long-term evolution experiment with *E. coli* for 50.000 generations [115]. Amongst others, most populations first evolved wider cells but later reverted to the length-to-width ratio of the ancestor. To optimize high-throughput screening methods, a nanowell platform has been developed to improve the resolution and throughput of microtiter plates [116]. As a single bacterium is inoculated per well, the genetic changes can be tracked easily [116]. Collectively, these findings demonstrate the feasibility of morphology engineering regulating key cell shape determinants.

Changing bacterial morphologies above a cellular level has also been investigated, namely the transition between a planktonic and biofilm lifestyle. In *Pseudomonas putida*, this process is regulated by controlling levels of cyclic di-GMP. Most interestingly, an artificial genetic system was designed to form biofilms at the user's will based on the heterologous expression of a diguanylate cyclase or a c-di-GMP phosphodiesterase under the control of a cyclohexanone-responsive expression system [117].

Despite the significant potential of morphology engineering, it is surprising that bacterial morphologies have only rarely been used for engineering purposes. Perhaps one of the primary reasons is that such strategies have so far only resulted in step-by-step improvements. In addition, many cell shape determinants are essential and thus difficult to engineer. Therefore, novel radical approaches are needed to dramatically redesign cell morphology, particularly in multicellular bacteria.

## 6 L-forms as an innovative platform for morphology engineering

**Fig. 3:**
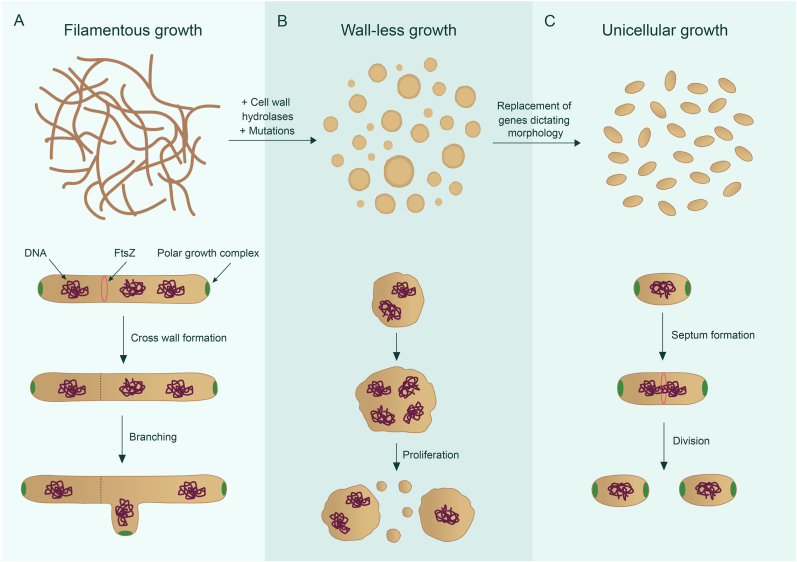
**Proposed innovative approach for morphology engineering of streptomycetes via L-forms.** Morphology of a filamentous streptomycete and its normal mode-of growth (A). Without their cell wall, spherical cells are obtained that proliferate without the canonical cell division machinery as so-called L-forms (B). These L-forms offer an innovative avenue for strain engineering, wherein the entire cell division apparatus can be substituted, such as with that of the unicellular bacterium *Corynebacterium*. This process holds the potential to create a unicellular *Streptomyces* strain (C).

Changing the morphology of a cell using canonical approaches is only possible to a certain extent: when genes required for cell division are removed beyond a minimal subset, this results in a combination of residual genes that does not allow the cell to survive. This contrasts with so-called L-forms, which are bacteria that proliferate in the complete absence of a cell division machinery [[118], [119], [120], [121]]. Instead, L-form division occurs due to an imbalance in the cell's surface-to-volume ratio caused by increased membrane synthesis, resulting in membrane blebbing and vesiculation [115,116]. To convert a walled bacterium into a successfully proliferating wall-deficient cell, the cell must escape from the sacculus, which can be achieved by adding hydrolases that degrade PG [122]. This process can also be stimulated by mutations that damage the cell wall structure [123]. Cells lacking a cell wall must also manage increased oxidative stress, often alleviated by mutations that lower metabolic flux through the TCA cycle, thereby reducing the production of reactive oxygen species [124]. Although L-forms have been challenging to isolate and cultivate, research on them has been gaining attention and showing promising results. L-forms can be obtained in a wide range of bacteria [[125], [126], [127], [128]], including streptomycetes (Fig. 3).

L-forms have already been shown to be a promising platform for producing biotechnological products, as a wall-less state can address issues related to incorrect folding, inclusion body formation, and cellular toxicity [129]. For instance, *Proteus mirabilis* L-forms have been utilized to increase the production of activable bovine prochymosin [130]. One reason for increased production could be the increased surface area for protein secretion in L-forms [131]. Besides, the cell wall can strongly affect the folding and stability of secreted proteins, especially when these are produced heterologously [132]. L-forms have also been proposed for the production of hydrophobic molecules because of their excess membrane, as membrane surface area can be a limiting factor for the accumulation of hydrophobic small molecules [131]. One other potential application of L-forms in the production of therapeutic proteins or peptides, as PG can trigger various innate immune responses, current downstream processes are costly and time-consuming [131]. L-forms have also been used to produce custom-made, synthetic phages [133]. In this case, its main advantage is the absence of the cell wall which otherwise would hamper the transfer of viral DNA into the cells. While production of metabolites in L-forms is possible, such cells are in most circumstances often not robust enough for large-scale industrial fermentations and would simply lyse due to high shear. Besides, L-forms often show a much lower growth rate. This problem could supposedly be solved by first accumulating some biomass of cells, before turning the appropriate genes on or off to switch to a wall-less state [131].

Like in other L-form systems, cell division genes could be removed in streptomycetes L-forms without noticeable effects on the proliferation of such cells [121]. For *Kitasatospora viridifaciens*, it has already been shown that the *divIVA* gene, as well as a larger part of the *dcw* cluster, can be deleted in L-forms [121], which is in line with other L-form systems [119]. As such, L-forms thus provide an unprecedented opportunity to redesign morphology radically (Fig. 3). To overcome the morphological constraints associated with filamentous growth, *Streptomyces* L-forms (Fig. 3B) could be modified by replacing the original cell division apparatus (Fig. 3A) with one sourced from a bacterium displaying a distinct, unicellular morphology, such as *C. glutamicum* (Fig. 3C). The rationale behind this choice is based on three reasons. First, *C. glutamicum* is also an actinobacterium with an apical growth machinery [134]. This is important as polar growth contributes to other crucial cellular processes, such as chromosome segregation [135,136]. Secondly, the cell division machinery of *C. glutamicum* is relatively simple in terms of structural composition compared to those of *E. coli* and *B. subtilis.* For instance, *C. glutamicum* lacks many regulatory systems (e.g. the well-studied Min system) that control the placement of the cell division site in unicellular bacteria such as *E. coli* and *B. subtilis* [137]. Third, we know that streptomycetes can grow with a cell wall even if a working, intact cell division machinery is not available: it is one of the rare examples where even FtsZ is not essential for survival [138]. However, this engineering process is complex and challenging due to differences in, amongst others, genetics, regulation, and slow growth. It would require a deep understanding of both donor and recipient systems, precise genetic manipulation techniques, and thorough testing to ensure the proper function and viability of the engineered cells.

## 7 Conclusion

As the potential of morphology engineering is enormous, bacterial morphologies could be manipulated further to enhance various metabolic engineering efficiencies. L-forms could become a valuable addition to current synthetic biology approaches to redesigning cell morphology radically. The expression of non-native cell division machineries would not only significantly improve our knowledge of the factors controlling cell morphology but it could also strongly optimize industrial production processes in recalcitrant bacteria. Besides these applied potentials, converting a multicellular, syncytial organism into a unicellular organism and vice-versa would allow us to tackle research questions that will broadly impact research fields beyond that of morphogenesis. For instance, ecological and evolutionary questions related to the functioning of such synthetic, non-filamentous cells within a colony or in association with other organisms are fascinating to address.

## CRediT authorship contribution statement

**Maarten Lubbers:** Writing – review & editing, Writing – original draft, Conceptualization. **Nova Jaspers:** Writing – original draft, Conceptualization. **Dennis Claessen:** Writing – review & editing, Supervision, Resources, Project administration, Funding acquisition, Conceptualization.

## Declaration of competing interest

The authors declare that they have no known competing financial interests or personal relationships that could have appeared to influence the work reported in this paper.
